# Task Difficulty Regulates How Conscious and Unconscious Monetary Rewards Boost the Performance of Working Memory: An Event-Related Potential Study

**DOI:** 10.3389/fnsys.2021.716961

**Published:** 2022-01-13

**Authors:** Shiyang Xu, Senqing Qi, Haijun Duan, Juan Zhang, Miriam Akioma, Fei Gao, Anise M. S. Wu, Zhen Yuan

**Affiliations:** ^1^Faculty of Health Sciences, University of Macau, Taipa, Macao SAR, China; ^2^Centre for Cognitive and Brain Science, University of Macau, Shanghai, Macao SAR, China; ^3^Guangdong-Hong Kong-Macao Greater Bay Area Center for Brain Science and Brain-Inspired Intelligence, Taipa, Macao SAR, China; ^4^Key Laboratory of Modern Teaching Technology, Ministry of Education, Shaanxi Normal University, Xi’an, China; ^5^Faculty of Arts and Humanities, University of Macau, Taipa, Macao SAR, China; ^6^Faculty of Social Sciences, University of Macau, Taipa, Macau SAR, China; ^7^Faculty of Education, University of Macau, Taipa, Macau SAR, China

**Keywords:** N-back task, working memory, P3, monetary reward, task difficulty level

## Abstract

The performance of working memory can be improved by the corresponding high-value vs. low-value rewards consciously or unconsciously. However, whether conscious and unconscious monetary rewards boosting the performance of working memory is regulated by the difficulty level of working memory task is unknown. In this study, a novel paradigm that consists of a reward-priming procedure and N-back task with differing levels of difficulty was designed to inspect this complex process. In particular, both high-value and low-value coins were presented consciously or unconsciously as the reward cues, followed by the N-back task, during which electroencephalogram signals were recorded. It was discovered that the high-value reward elicited larger event-related potential (ERP) component P3 along the parietal area (reflecting the working memory load) as compared to the low-value reward for the less difficult 1-back task, no matter whether the reward was unconsciously or consciously presented. In contrast, this is not the case for the more difficult 2-back task, in which the difference in P3 amplitude between the high-value and low-value rewards was not significant for the unconscious reward case, yet manifested significance for the conscious reward processing. Interestingly, the results of the behavioral analysis also exhibited very similar patterns as ERP patterns. Therefore, this study demonstrated that the difficulty level of a task can modulate the influence of unconscious reward on the performance of working memory.

## Introduction

Human beings tend to adjust their behaviors according to the rewards they might gain. To date, considerable studies have been carried out on human reward system, indicating that rewards can improve the performance of cognitive functions, such as working memory (Bijleveld et al., 2011; Capa et al., 2013), conflict monitoring (van Gaal and Lamme, 2012), bias responses (Garofalo et al., 2020), self-face processing (Zhan et al., 2017), and cognitive inhibition (Diao et al., 2016). Drawing on an incentive force task, Pessiglione et al. (2007) inspected the activation of the brain to the unconscious monetary reward cues, which revealed that the motor function could be improved with higher amounts of unconscious monetary reward, as compared to that with lower ones. In addition, the theory of the stimulus-response relation (Skinner, 1965) also demonstrates that people are apt to enhance their cognitive capabilities intentionally to gain more rewards. For instance, a recent study (Frömer et al., 2021) found that cognitive control is determined by the expectations of reward and efficacy collectively in a Stroop task. Nevertheless, even though both the conscious and unconscious reward cues were found to somehow facilitate various cognitive functions, these studies employed differing task demands. In particular, the reward of participants depended on their force execution in the incentive force paradigm (Pessiglione et al., 2007), while the Stoop task taps into control allocation of people. Less is known whether the facilitation effect on cognitive task performance from conscious and unconscious monetary rewards is regulated by differing difficulty levels of a task. This issue warrants examinations and discussions by more fine-grained tasks.

In addition to fundamental cognitive functions, existing literature also sheds light upon the conscious/unconscious reward effect on executive function, including working memory, flexibility, and inhibition control. As a higher-order cognitive function, working memory is crucial in the execution of complex cognitive tasks, such as learning, reasoning, and decision-making (Baddeley, 1992, 2010; Curtis and Lee, 2010). Interestingly, behavioral studies showed that working memory updating (Morris and Jones, 1990) could be enhanced by the monetary reward information unconsciously (Capa et al., 2011; Zedelius et al., 2011, 2014; Bijleveld et al., 2012). For example, Capa and Bouquet (2018) found that the dissociable effects of conscious and unconscious rewards on executive control (operationalized by working memory updating) are affected by the degree of reward sensitivity of participants. Participants with higher reward sensitivity demonstrated stronger reward effect in the conscious (i.e., supraliminal) settings, as compared to the unconscious (i.e., subliminal) condition when they were performing a numerical memory updating task. Yet, those with intermediate sensitivity performed better with high-value rewards regardless of the consciousness, whereas lower reward sensitivity was only related to a reward effect in the unconscious scenario. Moreover, Bijleveld et al. (2009) compared the difference in pupil dilation during the digit-retention task, suggesting that the difficulty level of a task exhibited a significant effect on the relationship between the value of unconscious reward and mental effort that participants involved. Another higher-order function is problem-solving. Cristofori et al. (2018) found that the unconscious and conscious reward motivational processes could influence the problem-solving that can be solved by deliberate insight. More importantly, the role of unconscious and conscious rewards on cognitive functions was also inspected, respectively. For example, Bijleveld et al. (2009) examined the performance of arithmetic problem-solving with high-value vs. low-value rewards consciously or unconsciously. They discovered that unconscious high rewards enabled participants to respond to the stimuli eagerly, whereas conscious rewards incurred participants to cautiously make a decision and concurrently respond more slowly and precisely.

Meanwhile, neuroimaging studies were also carried out to identify the neural indicators that were related to the processing of task preparation and execution underlying conscious or unconscious rewards (Coles, 1989; Leuthold and Jentzsch, 2002). For example, Capa et al. (2013) conducted an event-related potential (ERP) study, during which the preparatory process was inspected by using the response to the cue of a digit judgment task while subsequent task execution process was also assessed. They discovered that compared to task preparation, task execution was influenced differently by conscious and unconscious rewards. In particular, for the conscious reward trials, high-value rewards elicited higher P3 amplitudes in response to the task stimuli as compared to low-value rewards, indicating enhanced recruitment of attention and working memory resources. In contrast, this effect was not detected in the unconscious reward trials, demonstrating that conscious awareness of rewards uniquely affects task execution, independent of task preparation.

However, to date, the neural mechanism underlying the relationship between unconscious/conscious monetary reward and the execution task with different difficulty levels of a task remain unclear. Therefore, it is hypothesized in this study that the difficulty level might regulate the influence of unconscious and conscious reward on task execution. To test this hypothesis, electroencephalogram (EEG) recordings were carried out based on a new paradigm that consists of a classical N-back execution and a reward-priming task (Pessiglione et al., 2007). During the performance of this paradigm, participants were exposed to either high-value (1 yuan) or low-value (1 cent) monetary rewards, followed by several sequential N-back trials that involved the updating in working memory and restraining interference. It is expected that the difficulty level of a task can modulate the influence of unconscious reward on the performance of working memory. This study also paves a new avenue for advancing our understanding of the neural mechanism on how conscious and unconscious monetary rewards can boost the performance of working memory involving different difficulty levels.

## Materials and Methods

### Participants

A total of 34 right-handed undergraduates (20 women; mean age: 21.5) participated in this study. All participants with normal or corrected-to-normal vision were required to sign the informed consent documents before the experimental tests. The protocol for this study was approved by the Ethics Committees of the University of Macau. Participants with a history of neurological and psychiatric disorders were excluded from this study. The participants with extremely low or high scores on BIS/BAS scale would be excluded, thus leaving those with a score of 30 ± 5 (roughly intermediate level in reward sensitivity degree), in order to minimize the confounding effect of individual difference (Capa and Bouquet, 2018).

### Stimuli and Procedures

**FIGURE 1:**
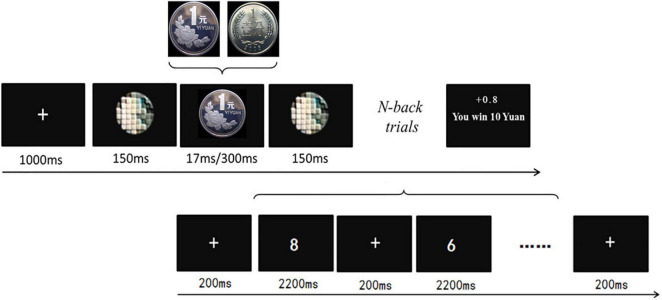
The procedure of the experiment. Each run consisted of 1 reward-priming trial and 17 N-back trials. One run started with a fixation cross (1,000 ms), followed by a pre-mask (150 ms), the reward stimulus (300 ms for the conscious vs. 17 ms for the unconscious stimuli), a post-mask (150 ms), and the digit N-back task with 17 trials. The total amounts of money they earned were displayed in the center of the screen.

This study drew on a within-participant design with reward values (1 cent for low value vs. 1 yuan for high value), reward types (300 ms for conscious vs. 17 ms for unconscious), and task difficulty (1-back for low difficulty vs. 2-back for high difficulty) as independent factors. A modified working memory paradigm was adopted, which consisted of a reward-priming task (Pessiglione et al., 2007) and a subsequent digital N-back task. As shown in Figure 1 for the experimental procedure, participants needed to perform the 1-back and 2-back tasks, respectively. Specifically, the yuan (Chinese: 元) is the basic unit of the Chinese currency (renminbi: RMB), and 1 yuan equals 100 cents. The 17-ms coin presentation corresponds to the unconscious reward condition, whereas the 300-ms coin presentation denotes the conscious reward condition.

Therefore, the present experimental design contained eight conditions: (1) 1-back task with high rewards presenting consciously, (2) 1-back task with low rewards presenting consciously, (3) 1-back task with high rewards presenting unconsciously, (4) 1-back task with low rewards presenting unconsciously, (5) 2-back task with high rewards presenting consciously, (6) 2-back task with low rewards presenting consciously, (7) 2-back task with high rewards presenting unconsciously, and (8) 2-back task with low rewards presenting unconsciously. Each condition included 8 runs; therefore, the paradigm consisted of 64 runs. All 64 runs were randomized and participants were able to take a rest every 4 runs. All stimuli were displayed in the center of the monitor with a 144-Hz refresh rate. E-prime software (Psychology Software Tools, Inc., Sharpsburg, PA, United States) was used to present the stimuli and collect the behavioral data. In this study, participants performed a practice session before the formal test. The practice session included 40 N-back trials.

For the formal test, each run started with the reward-priming task (1 trial), which was followed by the N-back task (17 trials: either 1-back or 2-back). For the reward-priming task, each trial contained the presentations of the fixation cross (1,000 ms), a pre-mask (150 ms), the reward stimulus (17 vs. 300 ms), and a post-mask (150 ms). As for the 1-back task of 17 consecutive trials, the first trial started with a fixation for 200 ms, and then a one-digit number (0–9) showed up (2,200 ms). After the first trial disappeared, the following trials included a fixation for 200 ms and then the probe number, where participants needed to make a response within 2,200 ms. They were asked to press the “b” key upon the computer keyboard as quickly and accurately once the number matched the right previous number, while they were to press the “n” key for the mismatch (see Figure 1). In addition, a jittered interval ranging from 500 to 1,000 ms was also added between any two 1-back trials. Similarly, the 2-back task required the participants to match the current number with the one 2 numbers backward.

During each run, participants were informed that the coin was either 1 cent or 1 yuan, which should be presented for a very brief period. Following the study by Bijleveld et al. (2014), when the accuracy rate (ACC) of participants of the 17 trials was lower than the earlier run, they received no money for this run. Besides, the amount of money they received was also influenced by the mean reaction time (RT) of the 17 trials. The shorter the RT, the more money the participants could gain. The amount of money the participants earned for each run was computed as follows:


(1) $$E=V-\left(V\times \frac{T}{A}\right)$$

where *E* is the amount of money one could earn, *V* is the value of the coin that was presented, and *T* is the mean RT of 17 N-back trials for each run. *A* was calculated as follows:


(2) A=2×(M⁢R⁢T+2×S⁢D⁢R⁢T)

where MRT and SDRT are, respectively, the mean and standard deviation of RT of the correct responses in the practice session.

Finally, participants received feedback individually, showing their ACC and the amount of money they earned for each run. The accumulated amounts of money they gained during the previous runs were also displayed on the monitor.

### Perceptual Discrimination Task

After the formal experiment, each participant was instructed to complete a forced-choice behavioral task to ensure that conscious monetary reward was consciously perceived while the unconscious monetary reward was not. At the beginning of each trial, a fixation was presented for 200 ms, followed by a pre-mask stimulus (300 ms), a reward stimulus (17 vs. 300 ms), and a post-mask stimulus (300 ms). And then participants needed to state what they saw by pressing the “m” key for 1 yuan and the “b” key for 1 cent. There were five practice trials and 60 formal trials, including 30 trials for 1 yuan and the other 30 trials for 1 cent case. During behavioral data collection, participants were told that accuracy was the most important objective in this session.

### Electroencephalogram Recordings and Preprocessing

Electroencephalogram data were acquired using a 64-channel Neuroscan system (Compumedics Neuroscan, Charlotte, NC, United States). The AFz channel upon the Ag/AgCl electrodes cap served as the ground channel, while the average of the two mastoid reference channels (i.e., M1 and M2) served as the reference. All inter-electrode impedances were kept below 5 kΩ and EEG data were digitalized with a sampling rate of 500 Hz.

EEGLAB (Delorme and Makeig, 2004) and ERPLAB (Lopez-Calderon and Luck, 2014) toolboxes were used for data processing and visualization. EEG data were first preprocessed by using a 0.1-Hz high-pass filter and a 30-Hz low-pass filter and then electrooculogram (EOG) artifacts were removed by using independent component analysis (Delorme and Makeig, 2004). Later, segments were extracted from epochs of -200 to 1,000 ms with a baseline correction of 200 ms prior to stimulus. In particular, the segments that contained voltage fluctuations exceeding ±100 V were discarded for further analysis. In addition, approximately 13.5% of the trials were excluded due to extensive artifacts, amplifier clipping, or peak-to-peak deflection.

## Data Analysis

### Behavioral Data

For behavioral data, the ACC reflected the degree to which mental effort was consumed during the N-back task while the mean RTs < 100 ms were eliminated for further analysis. In addition, a repeated-measures ANOVA was carried out on RT and ACC with reward values (1 cent vs. 1 yuan), reward types (unconscious reward vs. conscious reward), and difficulty levels of the task (1-back task vs. 2-back task) as within-subject factors. All repeated-measures ANOVAs were conducted with Greenhouse–Geisser correction if the sphericity assumption was violated, whereas *post-hoc* multiple comparisons were performed using Bonferroni-adjusted corrections. The performance of the perceptual discrimination task (i.e., ACC) was tested by using the independent sample *t*-test (*p* < 0.05) for each participant.

### Electroencephalogram Data

It is widely recognized that the parietal electrodes can exhibit large ERP component P3 (Capa et al., 2013; Diao et al., 2016). Therefore, the mean P3 amplitude of six parietal electrodes (i.e., CPz, CP1, CP2, P1, P2, and PZ) was inspected during 300–500 ms after trigger onset. Besides, a three-factor ANOVA on averaged P3 amplitude was performed with reward values (1 cent vs. 1 yuan), reward types (unconscious reward vs. conscious reward), and difficulty levels of the task (1-back task vs. 2-back task) as within-subject factors. All repeated-measures ANOVAs were conducted with Greenhouse–Geisser corrected if the sphericity assumption was violated, whereas *post-hoc* multiple comparisons were performed using Bonferroni-adjusted corrections. Effect sizes were presented as partial eta-squared ($\eta_{P}^{2}$) for *F* tests.

Pearson’s correction was used to analyze the relationship between the reward effect of the P3 amplitude and the ACC in the N-back task. The changes in the P3 amplitude and the ACC between high and low reward among all conditions were analyzed. The *p* values were corrected with the false discovery rate (FDR) correction.

## Results

### Behavioral Results

#### Accuracy Rate of N-Back Task

**FIGURE 2:**
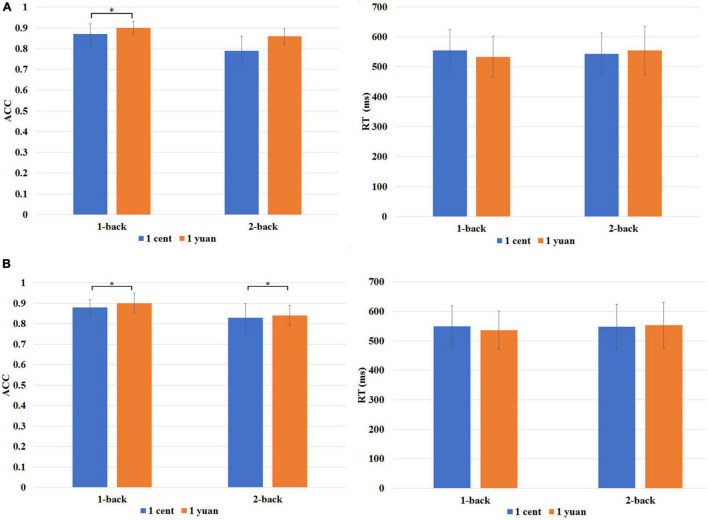
**(A)** Behavioral performance is associated with the high-value reward (1-yuan: orange bar) or low-value reward (1 cent: blue bar) under unconscious conditions. **(B)** Behavioral performance is associated with high- and low-value rewards under conscious conditions. RT denotes mean reaction time, while ACC denotes the accuracy rate of the N-back task. *p < 0.05.

The ACC of the N-back task was entered into a three-way repeated-measures ANOVA (i.e., reward value, reward type, and task difficulty). It was discovered that both the main effects of reward type [*F*(1, 33) = 36.88, *p* < 0.01, $\eta_{P}^{2}$ = 0.53] and difficulty levels of the N-back task [*F*(1, 33) = 77.92, *p* < 0.01, $\eta_{P}^{2}$ = 0.70] exhibited the significance. Further analysis showed that the interaction of three factors also reached statistical significance [*F*(1, 33) = 6.60, *p* < 0.05, $\eta_{P}^{2}$ = 0.17]. The simple effect analysis demonstrated that regarding 1-back task, participants performed more accurately in high-value rewards than that in low-value rewards for both unconscious [*F*(1, 33) = 21.58, *p* < 0.01, $\eta_{P}^{2}$ = 0.40] and conscious [*F*(1, 33) = 21.35, *p* < 0.01, $\eta_{P}^{2}$ = 0.39] conditions (refer to Figure 2A). However, this is not the case for the 2-back task. It was discovered that only for conscious condition, the ACC was significantly higher for high-value rewards as compared to that of low-value rewards [*F*(1, 33) = 20.95, *p* < 0.01, $\eta_{P}^{2}$ = 0.39]. In contrast, for unconscious condition, no significant difference between the high-value and low-value rewards was detected, *F*(1, 33) = 0.21, *p* > 0.05, $\eta_{P}^{2}$ = 0.01 (refer to Figure 2B).

#### Reaction Times of N-Back Task

A repeated-measures ANOVA was also carried out for the mean RTs of correct trials. The results indicated that the interaction effect of N-back difficulty level and reward value was significant [*F*(1, 33) = 8.91, *p* < 0.01, $\eta_{P}^{2}$ = 0.213]. The simple effect analysis showed that the average RT of high reward was shorter than that of low reward for the 1-back task [*F*(1, 33) = 5.39, *p* < 0.05, $\eta_{P}^{2}$ = 0.140]. It showed that participants need to take less time to complete the task for high-value reward as compared to that for low-value reward. However, no significant difference between the high reward and low reward was identified for the 2-back task, *F*(1, 33) = 2.37, *p* > 0.05, $\eta_{P}^{2}$ = 0.07 (refer to Figures 2A,B).

#### Visibility of Unconscious Reward

The mean ACC of unconscious perception (*M* = 49.11%, *SD* = 3%) approximated to chance level, indicating that participants were not consciously aware of the unconscious reward (*p* > 0.05, FDR-corrected). In contrast, the mean ACC (*M* = 81.47%, *SD* = 5%) was significantly different from 50% (*p* < 0.05, FDR-corrected), indicating that participants were not consciously aware of the unconscious reward.

### Event-Related Potential Results

**FIGURE 3:**
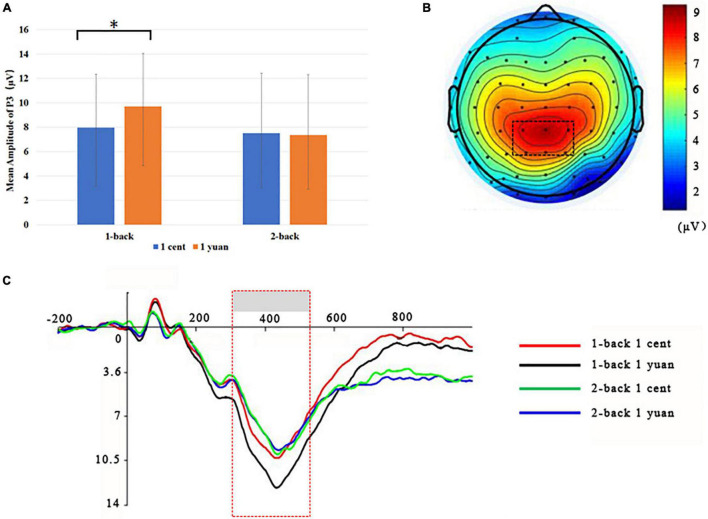
Grand-averaged ERPs for unconscious 1-back or 2-back task underlying the high- and low-value reward conditions. **(A)** High-value rewards elicited significantly larger P3 for unconscious 1-back trials and 2-back trials as compared to low-value rewards. *p < 0.05. **(B)** Scalp topographic maps for P3 amplitude with marked parietal ROI (region of interest). **(C)** Grand-averaged P3 for the 2-back task with the high-value reward (blue curve), the 2-back task with the low-value reward (green curve), the 1-back task with the high-value reward (black curve), and 1-back task with the low-value reward (red curve). The parietal ROI is the mean ERP from the electrodes CPz, CP1, CP2, P1, P2, and PZ with latency between 300 and 500 ms. The gray boxes highlighted the time windows for P3.

**FIGURE 4:**
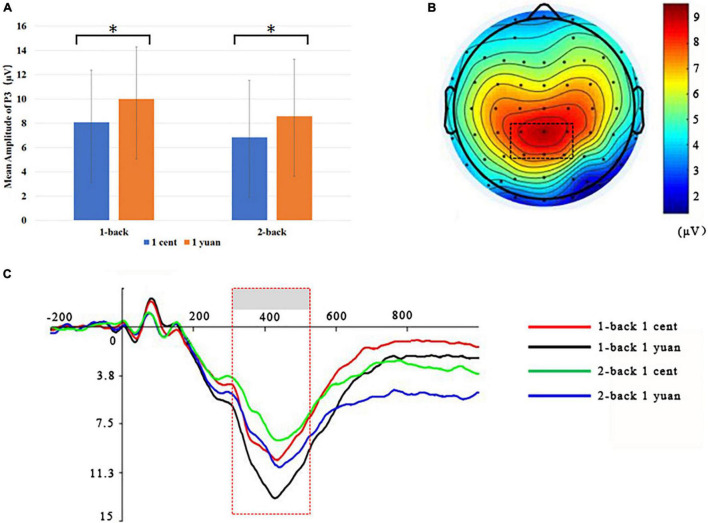
Grand-averaged ERPs for conscious 1-back and 2-back tasks underlying the high- and low-value reward conditions. **(A)** High-value rewards elicited greater P3 for conscious 1-back trials as compared to the low-value cases. *p < 0.05. **(B)** Scalp topographic maps for P3 amplitude with marked parietal ROI (region of interest). **(C)** Grand-averaged P3 for the 2-back task with the high-value reward (blue curve), a 2-back task with the low-value reward (green curve), the 1-back task with the high-value reward (black curve), and 1-back task with the low-value reward (red curve). The parietal ROI is the mean ERP from the electrodes CPz, CP1, CP2, P1, P2, and PZ with latency between 300 and 500 ms. The gray boxes highlighted the time windows for P3.

Electroencephalogram data demonstrated that the main effect of N-back difficulty level was significant [*F*(1, 33) = 11.24, *p* < 0.01, $\eta_{P}^{2}$ = 0.25]. In particular, the 1-back task elicited a larger P3 than the 2-back task. In addition, the main effect of reward type [*F*(1, 33) = 117.53, *p* < 0.01, $\eta_{P}^{2}$ = 0.50] also exhibited significance, demonstrating that high-value rewards induced larger P3 than low-value ones. Interestingly, it was discovered that the three-way interaction between the reward value, N-back difficulty, and reward type approached the level of significance [*F*(1, 33) = 7.49, *p* < 0.05, $\eta_{P}^{2}$ = 0.19]. To further inspect the three-way interaction involving the effect of reward type on the N-back task with different difficulty levels, the simple effect analysis was carried out. For 1-back task, the results demonstrated that high-value rewards exhibited larger P3 than low-value rewards for both unconscious [*F*(1, 33) = 9.20, *p* < 0.01, $\eta_{P}^{2}$ = 0.22] and conscious [*F*(1, 33) = 10.79, *p* < 0.01, $\eta_{P}^{2}$ = 0.25] conditions. However, for the 2-back task, enhanced P3 was detected for high-value rewards as compared to that from low-value rewards only when the reward value was consciously presented [*F*(1, 33) = 39.61, *p* < 0.01, $\eta_{P}^{2}$ = 0.55]. In contrast, when the reward value was presented unconsciously, P3 between the high and low rewards showed no significant difference [*F*(1, 33) = 2.17, *p* > 0.05, $\eta_{P}^{2}$ = 0.06; see Figures 3, 4 and Tables 1, 2].

**TABLE 1:** Behavioral data (in ms) and event-related potential (ERP) amplitudes (in μV) for unconscious 1-back and 2-back tasks underlying the high- and low-value reward conditions.

	1 cent		1 yuan	
	1-back	2-back	1-back	2-back
ACC	0.87 (0.05)	0.79 (0.07)	0.9 (0.03)	0.86 (0.04)
Parietal ROI (P3)	8.08 (4.28)	6.85 (4.69)	10.01 (4.95)	8.59 (4.97)
RT	554.02 (69.75)	543.52 (68.55)	533.14 (68.98)	554.1 (81.6)

*RT, reaction time; ROI, region of interest (CPz, CP1, CP2, P1, P2, and PZ).*

**TABLE 2:** Behavioral data (in ms) and ERP amplitudes (in μV) for conscious 1-back and 2-back tasks underlying the high- and low-value reward conditions.

	1 cent		1 yuan	
	1-back	2-back	1-back	2-back
ACC	0.88 (0.04)	0.83 (0.07)	0.9 (0.05)	0.84 (0.05)
Parietal ROI (P3)	7.98 (4.35)	7.5 (4.94)	9.7 (4.85)	7.37 (4.46)
RT	549.74 (69.46)	547.98 (76.38)	536.13 (65.71)	553.05 (76.94)

*RT, mean reaction time; ROI, region of interest (CPz, CP1, CP2, P1, P2, and PZ).*

### Correlations Between the Behavioral Data and the Event-Related Potential Data

**FIGURE 5:**
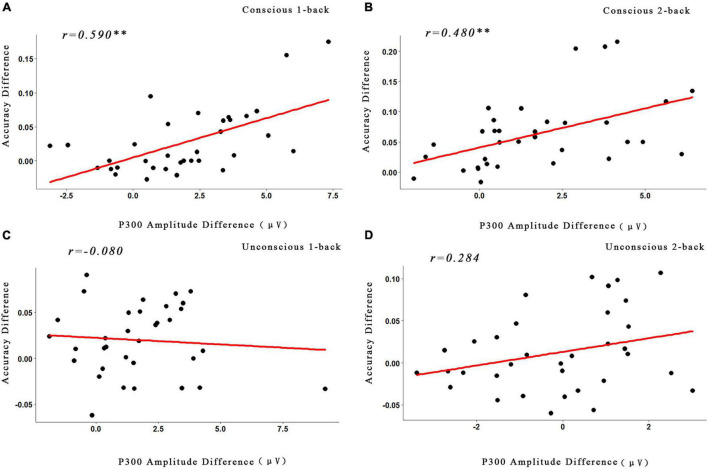
Correlations between the behavioral data and the ERP data. **(A)** Correlations between the changes of the P3 amplitude and the accuracy under the conscious 1-back task. **(B)** Correlations between the changes of the P3 amplitude and the accuracy under the conscious 2-back task. **(C)** Correlations between the changes of the P3 amplitude and the accuracy under the unconscious 1-back task. **(D)** Correlations between the changes of the P3 amplitude and the accuracy under the unconscious 2-back task. ^**^p < 0.01. The *p* values were corrected with the FDR correction.

The data demonstrated that the correlation between the ACC and P3 amplitude in the conscious reward conditions of 1-back (*r* = 0.590, *p* < 0.05, FDR-corrected) and 2-back (*r* = 0.480, *p* < 0.05, FDR-corrected) tasks was significant. In contrast, the correlation between the conscious reward conditions of 1-back (*r* = 0.41, *p* > 0.05, FDR-corrected) and 2-back (*r* = 0.41, *p* > 0.05, FDR-corrected) tasks was not significant (Figure 5).

## Discussion

The present behavioral and EEG neuroimaging studies inspected how the difficulty level in the N-back task can regulate the influence of unconscious and conscious rewards on task execution. In particular, higher ACC and enhanced P3 were detected for high rewards as compared to those from low rewards for both the unconscious and conscious priming conditions during the 1-back task. However, only the conscious condition for the 2-back task exhibited the same significance as that from the 1-back task. For the unconscious condition during the 2-back task, high-reward and low-reward priming exhibit no significant difference regarding the measures of ACC or P3. The interesting findings from the 1-back task showed good agreement with the Force Theory that human behaviors can be driven by the aims they pursue or the rewards they desire (Diao et al., 2016).

In addition, for the 2-back task, improved ACC in the high-reward case as compared to the low reward case was also identified when priming was consciously presented, although this effect was absent in the unconscious priming condition. Therefore, our pilot study demonstrated that the role of unconscious monetary reward on task execution might be regulated by the difficulty levels of the task. Furthermore, mean RTs results also exhibited significant interaction between the task difficulty level and reward values. During the 1-back task, participants responded faster to high rewards than that to low rewards. However, reward values showed no significant effect on the 2-back task, indicating that participants were motivated to respond fast in order to gain more rewards when the task was easy (1-back task). In contrast, they had to consume more time and mental efforts in order to respond accurately when the task was hard to complete (2-back task).

Further neural imaging data showed that high-value rewards exhibited larger P3 than low-value rewards when the task is easy (1-back task) or when the reward at stake is conscious for the 2-back task. However, no significant difference was identified between the high-value and the low-value rewards for the 2-back task when the reward is an unconscious case. These novel findings indicated that the difficulty level of the task to carry out might modulate the relationship between the task execution and the rewards consciously or unconsciously. Meanwhile, our behavioral and neural findings also validated that regarding our reward system, the conscious reward is in the charge of more advanced cognitive functions, including the organization of mental resources and processing of goal-related information. In particular, the unconscious reward has an essential influence on the performance of physical and simple tasks, especially when the reward is at stake and not intentionally perceived.

Taken together, these findings indicated that the task difficulty could modulate cognitive resources for the unconscious and conscious reward cues for priming working memory updating. This interpretation is in line with a previous study that unconscious motivation could affect high-order executive control functions (Pessiglione et al., 2007; van Gaal et al., 2008; Boy et al., 2010; Cristofori et al., 2018). Specifically, Capa et al. (2011) deployed an arithmetic updating task with the conscious and unconscious monetary reward. Their result suggested that the effect of the unconscious monetary would be absent when the task is difficult. Bijleveld et al. (2009) found that pupil dilation reflected the mental effort that the people did during the high-value reward task would be larger in harder tasks (five-digit), while it did not reach the level of statistical significance in easy tasks (three-digit). This study further validated the psychological reality of task difficulty in the interaction of reward consciousness and working memory by neuroimaging data.

However, the findings of this study showed that unconscious reward incentives did not modulate the difficult task, which could be attributed to the hierarchical mechanism of neural processing. In contrast to unconscious reward, when participants are aware of the reward intentionally, they could adopt their strategies to get that reward, especially in the higher-level cognitive functions. This implicates that conscious and unconscious reward effects might engage bottom-up and top-down processing strategies, respectively (Andrade et al., 2008; Lee and Shomstein, 2014). The conscious top-down reward effect is flexible, while the unconscious bottom-up reward effect is difficult to adjust for the task requirements. Moreover, in the view of human being evolution, reward system includes both the conscious and unconscious mechanisms, where the conscious system is in the charge of the advanced function, organizing more mental resources, and processing the goal-related information (Bijleveld, 2012; Bijleveld et al., 2012; Zhan et al., 2017). In contrast, the unconscious system has a critical influence on the task used to deal with automatic and perceptual operations. This feature could reallocate the mental resource economically, thus leaving more things for the necessities and gaining the chance to survive.

In addition to the behavioral and neuroimaging result, the correlation between the changes of P3 amplitude and the ACC was analyzed. According to the previous research method (Li et al., 2016; Thurm et al., 2018), we focused on the correlation between the difference of high and low reward of the P3 amplitude and ACC. As the results illustrated, the conscious reward (1-back and 2-back tasks) was significantly positively related between the two lines of results. However, the unconscious reward (1-back and 2-back tasks) did not show any significant correlation. This result also illustrated that the different pathways for the unconscious and conscious rewards promote cognitive ability, further suggesting that the executive function may be influenced only by conscious rewards, while the unconscious reward effect could not reach this stage (Thurm et al., 2018).

## Conclusion

We investigated the behavioral performance and neural activity of the N-back task of different difficulty levels induced by the conscious and unconscious monetary rewards. Our results indicated that people might invest more mental efforts and greater working memory in high-value rewards (unconscious and conscious) in the low-difficulty task. However, the interaction of the behavioral performance and the neural activity between conscious and unconscious monetary reward showed that the effect of unconscious reward on the high difficulty task was absent. The findings expand the insights into the relationship among the task demands, reward consciousness, and task execution and, thus, further revised the different functions of unconsciousness. Meanwhile, there is a limitation of this research that could be tackled in the future. Only the N-back task with the corresponding P3 component was used. Alternative paradigms with more ERP components of interest are expected in the future to enrich the scope of this topic.

## Data Availability Statement

The raw data supporting the conclusions of this article will be made available by the authors, without undue reservation.

## Ethics Statement

The studies involving human participants were reviewed and approved by University of Macau. The patients/participants provided their written informed consent to participate in this study.

## Author Contributions

HD, SQ, and SX conceived of the presented idea and carried out the experiments. ZY and SQ were involved in planning and supervised the work. ZY, SQ, FG, and SX wrote the manuscript. AW, JZ, FG, and MA revised the manuscript. All authors discussed the results and contributed to the final manuscript.

## Conflict of Interest

The authors declare that the research was conducted in the absence of any commercial or financial relationships that could be construed as a potential conflict of interest.

## Publisher’s Note

All claims expressed in this article are solely those of the authors and do not necessarily represent those of their affiliated organizations, or those of the publisher, the editors and the reviewers. Any product that may be evaluated in this article, or claim that may be made by its manufacturer, is not guaranteed or endorsed by the publisher.

## References

[B1] Andrade J. May J. Kavanagh D. (2008). Conscious and unconscious processes in human desire. *Psyche* 15 83–91.

[B2] Baddeley A. (1992). Working memory: the interface between memory and cognition. *J. Cogn. Neurosci.* 4 281–288. 10.1162/JOCN.1992.4.3.281 23964884

[B3] Baddeley A. (2010). Working memory. *Curr. Biol.* 20 R136–R140. 10.1016/J.CUB.2009.12.014 20178752

[B4] Bijleveld E. (2012). *The Unconscious and Conscious Foundations of Human Reward Pursuit*. *September 1983.* **CPQ.

[B5] Bijleveld E. Custers R. Aarts H. (2009). The unconscious eye opener: pupil dilation reveals strategic recruitment of resources upon presentation of subliminal reward cues. *Psychol. Sci.* 20 1313–1315. 10.1111/j.1467-9280.2009.02443.x 19788532

[B6] Bijleveld E. Custers R. Aarts H. (2011). Once the money is in sight: distinctive effects of conscious and unconscious rewards on task performance. *J. Exp. Soc. Psychol.* 47 865–869. 10.1016/j.jesp.2011.03.002

[B7] Bijleveld E. Custers R. Aarts H. (2012). Adaptive reward pursuit: how effort requirements affect unconscious reward responses and conscious reward decisions. *J. Exp. Psychol. Gen.* 141 728–742. 10.1037/a0027615 22468672

[B8] Bijleveld E. Custers R. Van der Stigchel S. Aarts H. Pas P. Vink M. (2014). Distinct neural responses to conscious versus unconscious monetary reward cues. *Hum. Brain Mapp.* 35, 5578–5586. 10.1002/hbm.22571 24984961 PMC4265283

[B9] Boy F. Husain M. Singh K. D. Sumner P. (2010). Supplementary motor area activations in unconscious inhibition of voluntary action. *Exp. Brain Res.* 206 441–448. 10.1007/S00221-010-2417-X 20871983

[B10] Capa R. L. Bouquet C. A. (2018). Individual differences in reward sensitivity modulate the distinctive effects of conscious and unconscious rewards on executive performance. *Front. Psychol.* 9:148. 10.3389/fpsyg.2018.00148 29503624 PMC5820315

[B11] Capa R. L. Bouquet C. A. Dreher J. C. Dufour A. (2013). Long-lasting effects of performance-contingent unconscious and conscious reward incentives during cued task-switching. *Cortex* 49 1943–1954. 10.1016/j.cortex.2012.05.018 22770561

[B12] Capa R. L. Bustin G. M. Cleeremans A. Hansenne M. (2011). Conscious and unconscious reward cues can affect a critical component of executive control. *Exp. Psychol.* 58 370–375. 10.1027/1618-3169/a000104 21310696

[B13] Coles M. G. H. (1989). Modern mind-brain reading: psychophysiology, physiology, and cognition. *Psychophysiology* 26 251–269. 2667018 10.1111/j.1469-8986.1989.tb01916.x

[B14] Cristofori I. Salvi C. Beeman M. Grafman J. (2018). The effects of expected reward on creative problem solving. *Cogn. Affect. Behav. Neurosci.* 18 925–931. 10.3758/s13415-018-0613-5 29949113 PMC6330050

[B15] Curtis C. E. Lee D. (2010). Beyond working memory: the role of persistent activity in decision making. *Trends Cogn. Sci.* 14 216–222. 10.1016/J.TICS.2010.03.006 20381406 PMC2883296

[B16] Delorme A. Makeig S. (2004). EEGLAB: an open source toolbox for analysis of single-trial EEG dynamics including independent component analysis. *J. Neurosci. Methods* 134 9–21. 10.1016/j.jneumeth.2003.10.009 15102499

[B17] Diao L. Qi S. Xu M. Li Z. Ding C. Chen A. (2016). Neural signature of reward-modulated unconscious inhibitory control. *Int. J. Psychophysiol.* 107 1–8. 10.1016/j.ijpsycho.2016.06.012 27346057

[B18] Frömer R. Lin H. Dean Wolf C. K. Inzlicht M. Shenhav A. (2021). Expectations of reward and efficacy guide cognitive control allocation. *Nat. Commun.* 12:1030. 10.1038/s41467-021-21315-z 33589626 PMC7884731

[B19] Garofalo S. Sagliano L. Starita F. Trojano L. di Pellegrino G. (2020). Subliminal determinants of cue-guided choice. *Sci. Rep.* 10:11926. 10.1038/s41598-020-68926-y 32681053 PMC7368056

[B20] Lee J. Shomstein S. (2014). Reward-based transfer from bottom-up to top-down search tasks. *Psychol. Sci.* 25:466. 10.1177/0956797613509284 24335604 PMC3933189

[B21] Leuthold H. Jentzsch I. (2002). Distinguishing neural sources of movement preparation and execution: an electrophysiological analysis. *Biol. Psychol.* 60 173–198. 12270590 10.1016/s0301-0511(02)00032-7

[B22] Li S. Du X. L. Li Q. Xuan Y. H. Wang Y. Rao L. L. (2016). ERP correlates of verbal and numerical probabilities in risky choices: a two-stage probability processing view. *Front. Hum. Neurosci.* 9:717. 10.3389/FNHUM.2015.00717 26834612 PMC4720736

[B23] Lopez-Calderon J. Luck S. J. (2014). ERPLAB: an open-source toolbox for the analysis of event-related potentials. *Front. Hum. Neurosci.* 8:213. 10.3389/fnhum.2014.00213 24782741 PMC3995046

[B24] Morris N. Jones D. M. (1990). Memory updating in working memory: the role of the central executive. *Bri. J. Psychol.* 81, 111–121. 10.1111/j.2044-8295.1990.tb02349.x

[B25] Pessiglione M. Schmidt L. Draganski B. Kalisch R. Lau H. (2007). How the brain translates money into force: a neuroimaging study of subliminal motivation. *Science* 26 157–165. 10.1016/0024-4937(90)90045-3 PMC2631941 17431137

[B26] Skinner B. F. (1965). *Science and Human Behavior (Issue 92904).* New York, NY: Simon and Schuster.

[B27] Thurm F. Zink N. Li S.-C. (2018). Comparing effects of reward anticipation on working memory in younger and older adults. *Front. Psychol.* 9:2318. 10.3389/FPSYG.2018.02318 30546333 PMC6279849

[B28] van Gaal S. Lamme V. A. F. (2012). Unconscious high-level information processing: implication for neurobiological theories of consciousness. *Neuroscientist* 18 287–301. 10.1177/1073858411404079 21628675

[B29] van Gaal S. Ridderinkhof K. R. Fahrenfort J. J. Scholte H. S. Lamme V. A. F. (2008). Frontal cortex mediates unconsciously triggered inhibitory control. *J. Neurosci.* 28:8053. 10.1523/JNEUROSCI.1278-08.2008 18685030 PMC6670759

[B30] Zedelius C. M. Veling H. Aarts H. (2011). Boosting or choking – How conscious and unconscious reward processing modulate the active maintenance of goal-relevant information. *Conscious. Cogn.* 20 355–362. 10.1016/j.concog.2010.05.001 20510630

[B31] Zedelius C. M. Veling H. Custers R. Bijleveld E. Chiew K. S. Aarts H. (2014). A new perspective on human reward research: how consciously and unconsciously perceived reward information influences performance. *Cogn. Affect. Behav. Neurosci.* 14 493–508. 10.3758/s13415-013-0241-z 24399682

[B32] Zhan Y. Xiao X. Chen J. Li J. Fan W. Zhong Y. (2017). Consciously over unconsciously perceived rewards facilitate self-face processing: an ERP study. *Sci. Rep.* 7:7836. 10.1038/s41598-017-08378-z 28798417 PMC5552778

